# Physicochemical, Pasting, and Thermal Properties of Native Corn Starch–Mung Bean Protein Isolate Composites

**DOI:** 10.3390/gels8110693

**Published:** 2022-10-26

**Authors:** Mohammad Tarahi, Fakhri Shahidi, Sara Hedayati

**Affiliations:** 1 Department of Food Science and Technology, Faculty of Agriculture, Ferdowsi University of Mashhad (FUM), Mashhad 9177948978, Iran; 2 Nutrition Research Center, School of Nutrition and Food Sciences, Shiraz University of Medical Sciences, Shiraz 7193635899, Iran

**Keywords:** mung bean protein isolate, corn starch, pasting properties, syneresis, retrogradation, MDPI, starch–protein mixture

## Abstract

Starch is widely used in food and non-food industries because of its unique characteristics. However, native starch shows some weaknesses that restrict its applications. Recently, some studies have demonstrated the benefits of using protein to overcome these limitations. Therefore, the aim of the present study was to investigate the effect of mung bean protein isolate (MBPI) (2%, 4%, 6%, and 8%) on the physicochemical, pasting, and thermal properties of native corn starch (NCS), as a novel starch–protein composite. Higher swelling power (SP), water absorbance capacity (WAC), and solubility values of NCS were observed with increasing MBPI concentration. Additionally, by the addition of MBPI, the rapid visco analyzer (RVA) showed a reduction in pasting temperature (77.98 to 76.53 °C), final viscosity (5762 to 4875 cP), and setback (3063 to 2400 cP), while the peak viscosity (4691 to 5648 cP) and breakdown (1992 to 3173 cP) increased. The thermal properties of NCS/MBPI gels investigated by differential scanning calorimetry (DSC) showed higher onset, peak, and conclusion temperatures (69.69 to 72.21 °C, 73.45 to 76.72 °C, and 77.75 to 82.26 °C, respectively), but lower gelatinization enthalpy (10.85 to 8.79 J/g) by increasing MBPI concentration. Fourier transform infrared spectroscopy (FT-IR) indicated that the addition of MBPI decreased the amount of hydrogen bonds within starch. Furthermore, after three cycles of freeze-thaw shocks, the syneresis of NCS-MBPI composites decreased from 38.18 to 22.01%. These results indicated that the MBPI could improve the physicochemical properties of NCS, especially its syneresis and retrogradation characteristics.

## 1. Introduction

Starch is a semi-crystalline high-molecular biopolymer that mainly consists of linear amylose and branched amylopectin chains [1]. It is one of the most abundant, inexpensive, and biodegradable polysaccharides, which is widely used in the food, pharmaceutical, and polymer industries to enhance the physicochemical properties of food and non-food products owing to its unique functional properties such as a thickening, stabilizing, gelling, and water retention [2,3]. Starch is obtained from varied sources, such as cereals, tubers, roots, legumes, and rhizomes [4]. About 80% of the world’s commercial production of starch is corn starch, so it is used in various products and applications [5]. However, native starch shows some weaknesses, including syneresis, retrogradation, and poor thermal, shear, and acid stability [6,7]. Hence, physical, chemical, and enzymatic modification methods are used to improve starch functionality. Because of the undesired processing materials that remain after chemical modification and the high price of enzymatic modification, recently, the tendency to use physical modification as a green and cheap method has increased [7,8]. Moreover, the incorporation of other food-derived ingredients such as proteins [9,10,11,12], polyphenols [13], and polysaccharides [6,14] to starch in order to improve its physicochemical properties has received much attention in recent years.

Proteins can improve the structural, textural, thermal, and pasting properties of food products through multiple interactions with other biopolymers, especially starch [15,16]. Moreover, the addition of protein can fortify starch-based products and make them healthier and more nutritionally balanced [17]. Numerous studies have been performed to study the interaction of animal-based proteins, especially milk proteins (whey protein and casein), with different starch sources. However, due to the environmental and ethical considerations, health concerns, and cost efficiency, the tendency of consumers and manufacturers has been shifted to plant-based foods and proteins [11]. Among plant-based proteins, interactions of legumes [15,18,19], wheat [20], corn [12,16], rice [21], and potato [22] proteins with starch have been studied during the past few years.

Legumes are dicotyledonous seeds, rich in carbohydrates (65 to 72%), proteins (18 to 32%), and dietary fibers (10 to 20%). Generally, they have remarkably higher protein content compared to cereal seeds, and are considered as important sources of proteins, essential amino acids, and bioactive peptides for large parts of the world’s population [11,18,23]. The application of legume proteins is usually restricted to soybean protein. For example, Chinma et al. [24] reported that the pasting temperature, peak viscosity, and final viscosity of cassava starch were significantly increased from 71.0 to 72.3 °C, 160.1 to 268.3 RVU, and 140.4 to 211.1 RVU, respectively, by adding different concentrations of soy protein. Additionally, Zheng et al. [12] represented lower setback (192.0 cP) and enthalpy (8.8 J/g) values for proso millet starch–soybean protein isolate mixtures compared to proso millet starch (343.0 cP and 15.2 J/g, respectively), which indicated better cold paste properties and less energy requirement for starch granule dissociation of the mixtures. However, studies should focus on exploration and utilization of proteins from alternative sources such as mung beans [11,18]. In addition, proteins can affect the pasting and thermal properties of starch-based food matrices in different ways, owning to their type and concentration, which indicates the necessity of evaluating each starch–protein system individually [25].

Mung bean (*Vigna radiata* L.) is a plant species from the legume (Fabaceae or Leguminosae) family, which is cultivated in almost 8.5% of the global legume area. Mung bean seed is a good source of micro- and macro-nutrients, such as carbohydrates, minerals, vitamins, dietary fibers, as well as anti-bacterial, anti-fungal, and bioactive components [26,27]. Moreover, it has a high protein content (20 to 27%), which can be used as a rich and inexpensive source of protein in the food industry [28,29]. The application of mung bean protein isolate (MBPI) has gained increasing attention in recent years not only due to its nutritional benefits, but also due to its favorable functional characteristics, such as excellent emulsifying, foaming, gelling ability, and water absorption properties [30,31]. Proteins with high water absorption capacity (WAC) can improve the water distribution in starch-based foods and modify the interactions between water molecules and other components, which leads to better textural stability [25]. Thus, MBPI can be used in a variety of starch-based food systems. In our previous study, we investigated the effects of MBPI on turbidity, syneresis, crystallinity, textural, structural, and rheological characteristics of native corn starch (NCS) [32]. In addition, to have a deeper understanding of the NCS-MBPI composites, the main objective of the present study was to investigate the effects of different concentrations (2%, 4%, 6%, and 8% dry starch weight basis) of MBPI on the swelling power (SP), solubility, and WAC values, as well as the freeze-thaw stability, Fourier transform infrared (FT-IR) spectra, pasting, and thermal properties of NCS.

## 2. Results and Discussion

### 2.1. Swelling Power, Water Absorption Capacity, and Solubility

**Figure 1:**
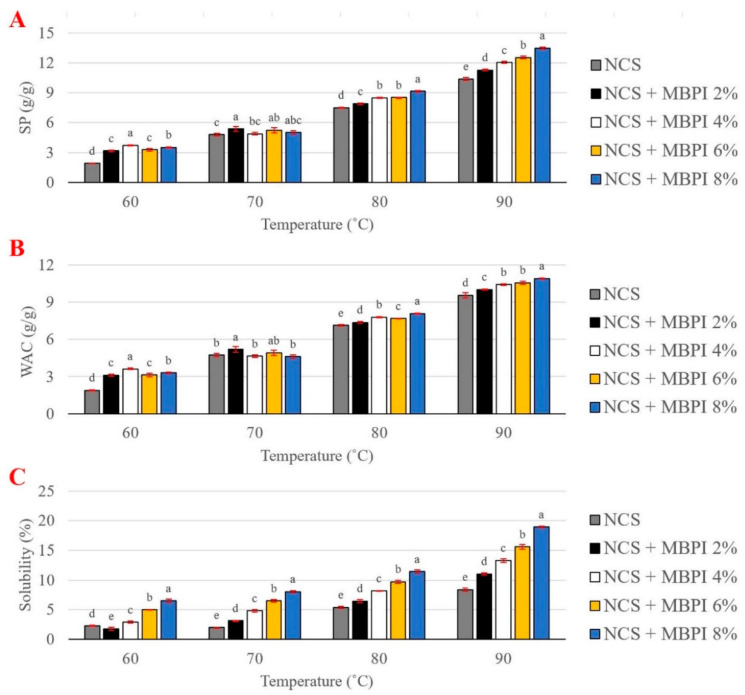
Swelling power (**A**); water absorption capacity (**B**); and solubility (**C**) of NCS and NCS/MBPI gels in different temperatures (60 °C, 70 °C, 80 °C, and 90 °C). Different letters at each temperature indicate significant differences (*p*) between treatments.

When the starch granules are heated in excess water, the crystalline structure is destroyed. Consequently, hydrogen bonds are formed between the hydroxyl groups of starch granule chains and water, which causes granule swelling [33]. The SP, WAC, and solubility of NCS and NCS/MBPI gels are shown in Figure 1. All parameters were temperature-dependent and increased with the increase in temperature, with the highest amount at 90 °C. The SP reflects the WAC of starch granules during gelatinization [34]. Both the SP and WAC values of NCS were increased by adding MBPI (Figure 1A,B). At 60 to 90 °C, the SP ranged from 1.92 to 3.52, 4.80 to 5.01, 7.51 to 9.15, and 10.38 to 13.47 (g/g). Moreover, the highest WAC was observed by adding 8% of MBPI to NCS, with a value of 10.92 g/g. Similar results were reported for adding soy protein concentrate to barley starch–lentil protein concentrate blends [9]. In contrast, Sun and Xiong [34] observed reducing SP and WAC of pea starch by blending it with pea protein isolate.

The hydration properties of starch–protein composite depend on the characteristics of its components, their ratio, and their interactions [19]. MBPI is deformed and denatured during heating; therefore, hydrophilic groups (such as –COOH, –SH, –NH_2_, and –OH) and hydrophobic amino acids (such as Leu, Ile, Met, Val, Ala, Gly, Phe, Tyr, and Trp) are exposed, which could form electrostatic or hydrogen bonds with other components in the system [27,35]. Higher WAC and SP of NCS/MBPI gels at 80 and 90 °C compared to NCS may be due to the protein’s hydroxyl groups (–COOH), which could bind the water molecules, and/or due to the presence of polar (hydrophilic) amino acids in MBPI, which could act as primary sites for interaction with water and starch molecules in the starch–protein system [36]. Additionally, Du et al. [31] reported that the WAC of MBPI was higher than that of fenugreek protein concentrates and soy protein isolate. The hydrophobic amino acids could reduce the WAC of starch–protein composite by covering the surface of starch granule and inhibiting the starch swelling [9,37]. However, the hydrophilic groups and amino acids could form crosslink bonds with water and/or starch and promote starch swelling [19]. Besides the importance of the protein surface hydrophilicity/hydrophobicity ratio, its amino acid composition, size, and molecular weight could affect the WAC of protein and starch–protein composite [9,31].

The solubility of NCS with and without the MBPI was shown in Figure 1C. The solubility value indicates the amylose leaching of starch granules among the gelatinization. Adding MBPI to NCS increased the solubility values significantly (*p* < 0.05). However, it should be mentioned that the MBPI is soluble in water, and these solubility values do not represent the actual amylose leaching of corn starch granules [17].

### 2.2. Pasting Properties

The pasting characteristics of NCS/MBPI gels are shown in Table 1. The addition of MBPI significantly affected the different pasting parameters of NCS (*p* < 0.05). The PT, SB viscosity, and FV of composites decreased (77.98 ± 0.02 to 76.53 ± 0.06 °C, 3063 ± 18 to 2400 ± 37 cP, and 5762 ± 15 to 4875 ± 28 cP) by increasing the MBPI concentration, while the PV and BD viscosity of the composites increased (4691 ± 17 to 5648 ± 13 cP and 1992 ± 20 to 3173 ± 19 cP). These results are similar to those of previous studies [36,38].

PT is the minimum temperature in which the viscosity starts to increase because of heating, which negatively correlates with the water binding capacity (WBC) of the system [34]. The decrease in PT of NCS/MBPI gels could be related to the increase in WAC of the starch–protein composite (Figure 1B). PV showed the highest swelling degree of gelatinized starch granules during heating. The gelatinized starch suspension is comprised of starch granules (dispersed phase) in an amylose/amylopectin aqueous solution (continuous phase) [36]. The viscosity of starch–protein systems is mainly determined by the interactions in the continuous phase, which prevent the granules’ implosion and increase the starch granules’ SP and PV [39]. The increase in PV of the starch after the addition of proteins may be due to the protein interactions with leached amylose [18], the interactions of hydrophilic protein sides with long side branches of amylopectin [38], and also protein self-aggregation and intermolecular interactions [40]. The increase in PV and SP (Section 2.1) of the starch–protein composite weakens the starch granule structure, which increases the BD viscosity during heating and shearing. These findings are consistent with previous studies [32,41].

The SB viscosity reflects the reaggregation of the swollen starch granules after cooling of the starch paste and indicates the short-term (amylose) retrogradation tendency [12]. The viscosity of starch paste after holding at 50 °C at the end of the RVA experiment is called FV [41]. As shown in Table 1, the SB viscosity and FV of starch–protein mixtures decreased with increasing MBPI concentration. In other words, the MBPI can retard the NCS retrogradation. Liang and King [42] reported that the negatively charged amino acids (such as Asp, Glu) could decrease the starch SB viscosity. The more negatively charged amino acids than positively charged ones (such as Arg) and the negative zeta-potential of MBPI may result in the formation of electrostatic interactions between protein polypeptides and NCS molecules and prevent rearrangement of amylose, which is responsible for the decrease in SB viscosity and FV [9]. Additionally, Niu et al. [5] reported the same results for adding the porcine plasma protein hydrolysates to corn starch.

### 2.3. Thermal Properties

The thermal parameters of the NCS/MBPI samples are shown in Table 2. The onset, peak, and conclusion transition temperatures (T_o_, T_p_, and T_c_) of composite gels increased with increasing the MBPI concentrations (69.69 ± 0.42 to 72.21 ± 0.20 °C, 73.45 ± 0.40 to 76.72 ± 0.15 °C, and 77.75 ± 0.35 to 82.26 ± 0.24 °C, respectively), while the gelatinization enthalpy (ΔH) decreased significantly (*p* < 0.05) from 10.85 ± 0.23 to 8.79 ± 0.15 J/g. The same results have been reported for other starch–protein composites [10,19]. However, these trends were opposed to the behavior of the pasting temperature (Section 2.2) measured by RVA. The gelatinization transition temperatures (T_o_, T_p_, and T_c_) are related to the internal crystalline structure of starch and its heat stability, and the ΔH reflects the starch crystallinity and the energy needed for double helical dissociation [35]. The system’s water content is important in determining the thermal parameters of starch, and lower water content causes an increase in gelatinization transition temperatures [10]. Therefore, the increase in gelatinization transition temperatures could be due to the reduced available water for NCS by adding MBPI [2]. Ding et al. [35] reported that the presence of proteins in starch–protein composites could increase the gelatinization transition temperatures of gelatinized starch by interfering with the water mobility and weakening the water-starch interactions. Additionally, Colombo et al. [40] confirmed that this phenomenon may be due to the interaction of the protein with leached amylose, resulting in the restriction of internal amylose and amylopectin combination. Consequently, MBPI could affect the NCS gelatinization process by its ability to retain water and interact with starch molecules.

### 2.4. FT-IR Spectroscopy Analysis

**Figure 2:**
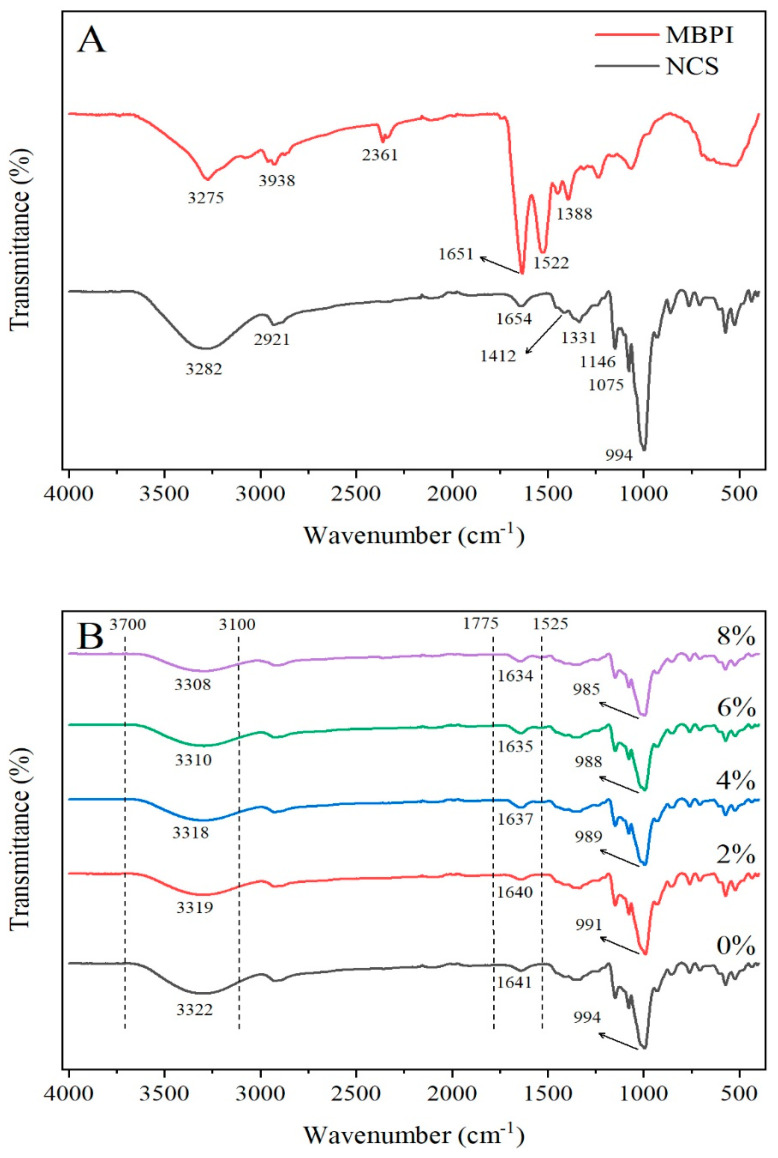
FT-IR spectra of NCS and MBPI (**A**) and NCS/MBPI gels, (**B**) at different MBPI concentrations (2%, 4%, 6%, and 8%).

FT-IR spectrometry was used to evaluate the feasibility of the interactions between starch and protein. Spectra of NCS and MBPI powders and the NCS/MBPI gels are presented in Figure 2A and 2B, respectively. The sharp peak at a wavenumber of 1651 cm^−1^ in the FT-IR spectra of MBPI corresponded to the C=O stretching, which may be related to the amide I region. Additionally, the peaks at wavenumbers of 1500 to 1580 cm^−1^ (amide II) corresponded to the C–H stretching and N–H bending vibrations, and 1200 to 1400 cm^−1^ (amide III) corresponded to the complex mix of α-helix and β-helix. These regions represent the secondary and tertiary structures of proteins [43]. Moreover, the peaks at 3275 cm^−1^ and 2938 cm^−1^ might correspond to the overlapping of O–H and N–H stretching vibrations and C–H stretching vibrations, respectively [44]. NCS showed the main absorption bands at 3282, 2921, 1654, 1331, and 994 cm^−1^, which were related to the O–H stretching, C–H group, water adsorption, angular twisting of CH_2_, and stretching of the glucose ring, respectively [3,5,6,10]. The bond at 1412 cm^−1^ corresponded to –COOH [5]. The bonds at 1146 cm^−1^ and 1075 cm^−1^ were related to the C=O group and CH_2_–OCH_2_ stretching vibrations [3]. Compared with NCS, the spectra of the NCS/MBPI composite represented no new absorption peak, indicating that there was no covalent binding formed between NCS and MBPI during pasting (Figure 2B). The band at 3322 cm^−1^ was reduced to a wavenumber of 3308 cm^−1^ after adding 8% MBPI, which indicated the weaker strength of hydrogen bonds and lower retrogradation [5]. Moreover, the peaks of the mixture at 3100 to 3700 cm^−1^ and 1525 to 1775 cm^−1^ became wider and redshifted with higher concentrations of MBPI, which were related to forming the intermolecular hydrogen bonds between MBPI and the amorphous region of the starch molecule [6]. Additionally, this phenomenon indicated that stronger hydrogen bonds formed between MBPI and leached amylose than between amylose themselves, which caused lower amylose recrystallization and decreased the short-term retrogradation of starch [5,14].

### 2.5. Freeze-Thaw Stability

**Figure 3:**
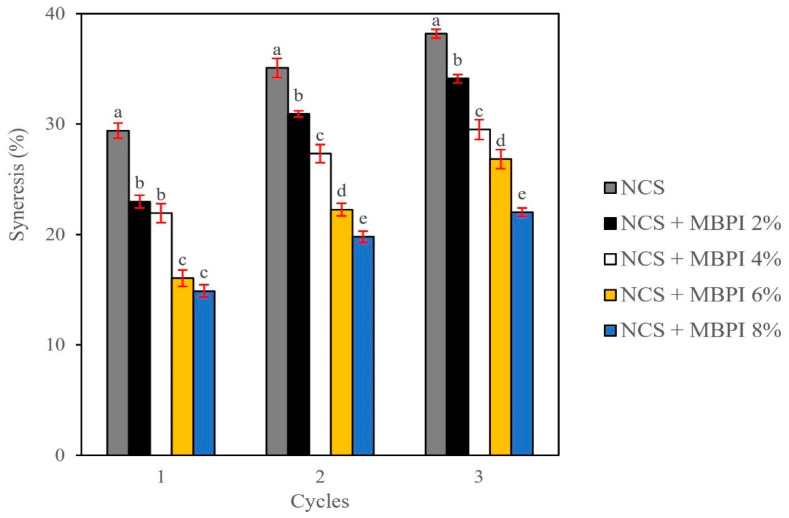
The freeze-thaw stability of NCS and NCS/MBPI gels in three cycles. Different letters in each cycle indicate significant differences between treatments.

The ability of starch to overcome physical changes during temperature shocks is estimated using freeze-thaw stability [45]. Syneresis is the release of water from a starch gel network due to the rearrangement of the leached starch molecules. It is an undesirable phenomenon and can be used as an indication of starch retrogradation [17]. The syneresis of NCS and NCS/MBPI gels is shown in Figure 3. The syneresis of NCS gels was decreased by the addition of MBPI, and the higher concentrations of MBPI improved the stability of NCS gels more efficiently. The addition of 8% MBPI could lower the syneresis of NCS gels from 29.41 to 14.88%, 35.08 to 19.80%, and 38.18 to 22.01% during freeze-thaw cycles. Previously, Colombo et al. [40] reported that the soybean protein concentrate could decrease the syneresis of corn and wheat starch gels. In addition, Niu et al. [5] showed that the porcine plasma protein hydrolysates decreased the corn starch retrogradation due to the protein interactions with leached amylose and also because of its water absorption. Moreover, Chen et al. [46] showed a reduction in the syneresis of potato starch gel after adding modified glutenin and gliadin due to its hydrogen bond interaction or chain involvement with amylose. In contrast, Anbarani et al. [45] and Kumar et al. [17] reported that the syneresis of starch gels was increased by its substitution with whey protein concentrate, which could be attributed to the lower starch fraction that made the starch network weak. Therefore, lower syneresis of NCS/MBPI gels may be due to the MBPI capability of holding more water in the system and also its interaction with leached amylose, which could retard the amylose rearrangement and decrease the syneresis and retrogradation of NCS starch gel. This observation is in line with our other results (Section 2.2, Section 2.3, Section 2.4).

## 3. Conclusions

This study showed that the MBPI could interact with the leached amylose by forming intermolecular hydrogen bonds during the gelatinization, which could disturb the amylose–amylose interaction and retard its rearrangement and retrogradation. Additionally, the presence of MBPI could enhance the WBC of the system and decrease the syneresis, especially at higher concentrations. Overall, these results suggest that the addition of MBPI to NCS could significantly improve the physicochemical and functional properties of NCS, which could prolong the shelf-life of starchy food products by retarding their short-term retrogradation and reducing the syneresis.

## 4. Materials and Methods

### 4.1. Materials

Mung bean seeds were purchased from a local market in Shiraz, Iran. Native corn starch was obtained from the Behinazma company (Shiraz, Iran). All chemical materials were purchased from Sigma Chemical Co. (St Louis, MO, USA) and were of analytical grade.

### 4.2. Isolation of Mung Bean Protein

The protein isolates were prepared using the alkaline extraction precipitation process described by El-Adawy [30] with some modifications. First, mung bean seeds were cleaned by removing foreign matters, washed, and then milled into the fine flour using an electrical miller (model OE-830 Olympia, Glarus, Switzerland) and passed through a sieve (120 mesh no.). Then, 20 g of mung bean flour was added to distilled water (1:10 *w/v*), and the pH of the dispersion was adjusted to 9 by NaOH. Then, it was stirred for 1 h using a magnetic stirrer at 25 °C and centrifuged for 6 min at 3000× *g*. After that, the pH of the supernatant was adjusted to pH 4.5 with HCl according to precipitate the protein and then centrifuged at 3000× *g* for 6 min and then collected. The protein curd was washed three-times with distilled water and neutralized by NaOH before freeze-drying. Finally, the protein content of MBPI was determined using the micro-Kjeldahl technique [47], which was 89%.

### 4.3. Preparation of NCS/MBPI Blends

MBPI was mixed with NCS at different concentrations (0%, 2%, 4%, 6%, and 8% of NCS dry weight) using a mixer (PARS KHAZAR, BG-300P, Rasht, Iran).

### 4.4. Swelling Power (SP), Water Absorption Capacity (WAC), and Solubility

The SP, WAC, and solubility values of the blend samples (see Section 4.3) were determined by the methods of Tarahi et al. [48] and Sun and Xiong [34]. First, 40 mL of distilled water was poured into pre-weighted centrifuge tubes and 0.4 g of each sample was dispersed in the water. After that, the dispersions were heated at 60 °C, 70 °C, 80 °C, and 90 °C under continuous stirring for 30 min in a water bath. The samples were cooled down to ambient temperature (25 °C) before being centrifuged for 5 min at 6000× *g*. The supernatant was poured into a pre-weighted Petri dish and dried overnight at 55 °C. The WAC, solubility, and SP of the samples were determined by the following Equations (1)–(3):

WAC (g/g) = weight of swollen granules/weight of sample(1)

Solubility (%) = (weight of dried supernatant/weight of sample) × 100(2)

SP (g/g) = weight of swollen granules × 100/weight of sample × (100 − % solubility)(3)

### 4.5. Rapid Visco Analyzer (RVA) Measurements

Pasting properties were determined using the RVA (Starch Master2, Perten, Macquarie Park, Australia). For this experiment, 25 mL of distilled water and 3.5 g of NCS-MBPI mixture were poured into an aluminum canister and stirred manually by the plastic paddle of the instrument for 60 s to assure the sample was fully dispersed in distilled water. The slurry was subjected to a programmed 13.0 min heating and cooling cycle, where the sample was held at 50 °C for 1 min, heated to 95 °C at a rate of 5 °C/min, held at 95 °C for 2.7 min, cooled down to 50 °C at a rate of 5 °C/min, and finally held at 50 °C for 2 min. The stirring speed of the paddle for the first 10 s was 960 rpm, and for the rest of the experiment was 160 rpm; then, the pasting parameters, including pasting temperature (PT), peak viscosity (PV), final viscosity (FV), setback (SB), and breakdown (BD) were calculated, and the viscosity was measured in centipoise (cP).

### 4.6. Differential Scanning Calorimeter (DSC)

The thermal properties of the blend samples were examined according to the method of Zheng et al. [12], using a DSC (Mettler Toledo TGA/DSC1, Greifensee, Switzerland). The samples (~3 mg, dsb) were mixed with 10 mL of distilled water in the aluminum pan, sealed, and equilibrated overnight. They were heated from 25 to 150 °C at a rate of 10 °C/min to evaluate the thermal parameters, such as transition temperatures (T_o_, T_p_, and T_c_) and gelatinization enthalpy (ΔH).

### 4.7. Fourier Transform Infrared (FT-IR) Spectroscopy

The pasted samples from the RVA analysis (Section 4.5) were freeze-dried and their FT-IR spectra were obtained by a spectrometer (Tensor II, Bruker, Ettlingen, Germany) at room temperature. The FT-IR spectra was determined in the range of 4000 to 400 cm^−1^ with a resolution of 4 cm^−1^.

### 4.8. Evaluation of Freeze-Thaw Stability

The freeze-thaw properties of the blend samples were evaluated by the method of Hoover and Senanayake [49] with a modification of Anbarani et al. [45]. First, 10 g of the blended samples (see Section 2.3) were placed in 50 mL tubes and gelatinized in a water bath at 95 °C for 30 min (under continuous stirring). After cooling at room temperature, the tubs were stored at −20 °C for one day (24 h), and thawed for three hours in a water bath (30 °C). Then, the samples were centrifuged for 10 min at 8000× *g*. These freeze-thaw cycles were repeated for three cycles, and the syneresis was determined after each cycle by the following Equation (4):

Syneresis (%) = Weight of released-liquid/Weight of initial sample × 100(4)

### 4.9. Statistical Analyses

The analysis of variance (ANOVA) and Duncan’s multiple range test were used to study the differences between the mean values at a 5% significance level, using SPSS software, version 26 (IBM Company, Chicago, IL, USA). All experiments were performed in triplicate.

## Figures and Tables

**Table 1:** Pasting properties of NCS/MBPI gels at different MBPI concentrations.

MBPI (%)	Pasting Temperature (°C)	Viscosity (cP)			
		Peak	Breakdown	Setback	Final
0	77.98 ± 0.02 ^a^	4691 ± 17 ^e^	1992 ± 20 ^e^	3063 ± 18 ^a^	5762 ± 15 ^a^
2	77.06 ± 0.03 ^b^	5002 ± 14 ^d^	2397 ± 25 ^d^	2738 ± 37 ^b^	5343 ± 25 ^b^
4	77.04 ± 0.01 ^b^	5282 ± 22 ^c^	2685 ± 22 ^c^	2612 ± 18 ^c^	5209 ± 13 ^c^
6	76.86 ± 0.06 ^c^	5578 ± 10 ^b^	3049 ± 23 ^b^	2475 ± 44 ^d^	5004 ± 12 ^d^
8	76.53 ± 0.06 ^d^	5648 ± 13 ^a^	3173 ± 19 ^a^	2400 ± 37 ^e^	4875 ± 28 ^e^

Means ± standard deviations of triplicate analysis. Means in the same column with different letters are significantly different (*p* < 0.05).

**Table 2:** Thermal properties of NCS and NCS/MBPI gels at different MBPI concentrations.

MBPI (%)	Thermal Parameters			
	T_o_ (°C)	T_p_ (°C)	T_c_ (°C)	ΔH (J/g)
0	69.69 ± 0.42 ^e^	73.45 ± 0.40 ^e^	77.75 ± 0.35 ^d^	10.85 ± 0.23 ^a^
2	70.43 ± 0.12 ^d^	74.71 ± 0.19 ^d^	80.78 ± 0.20 ^c^	9.77 ± 0.07 ^b^
4	71.24 ± 0.12 ^c^	75.36 ± 0.24 ^c^	81.17 ± 0.17 ^bc^	9.54 ± 0.11 ^b^
6	71.69 ± 0.14 ^b^	76.10 ± 0.16 ^b^	81.43 ± 0.19 ^b^	9.11 ± 0.09 ^c^
8	72.21 ± 0.20 ^a^	76.72 ± 0.15 ^a^	82.26 ± 0.24 ^a^	8.79 ± 0.15 ^d^

Means ± standard deviations of triplicate analysis. Means in the same column with different letters are significantly different (*p* < 0.05).

## Data Availability

The data presented in this study are available upon request from the corresponding author.
